# MobileYOLO: Real-Time Object Detection Algorithm in Autonomous Driving Scenarios

**DOI:** 10.3390/s22093349

**Published:** 2022-04-27

**Authors:** Yan Zhou, Sijie Wen, Dongli Wang, Jiangnan Meng, Jinzhen Mu, Richard Irampaye

**Affiliations:** 1 School of Automation and Electronic Information, Xiangtan University, Xiangtan 411105, China; wensijie0711@163.com (S.W.); wangdl@xtu.edu.cn (D.W.); mjnshizhu@163.com (J.M.); 2 Shanghai Aerospace Control Technology Institute, Shanghai 201109, China; jinzhen_mu@163.com; 3 School of Mathematics and Computational Science, Xiangtan University, Xiangtan 411105, China; richarcive@gmail.com

**Keywords:** autonomous driving, object detection, real-time, YOLOv4, KITTI data set

## Abstract

Object detection is one of the key tasks in an automatic driving system. Aiming to solve the problem of object detection, which cannot meet the detection speed and detection accuracy at the same time, a real-time object detection algorithm (MobileYOLO) is proposed based on YOLOv4. Firstly, the feature extraction network is replaced by introducing the MobileNetv2 network to reduce the number of model parameters; then, part of the standard convolution is replaced by depthwise separable convolution in PAnet and the head network to further reduce the number of model parameters. Finally, by introducing an improved lightweight channel attention modul—Efficient Channel Attention (ECA)—to improve the feature expression ability during feature fusion. The Single-Stage Headless (SSH) context module is introduced to the small object detection branch to increase the receptive field. The experimental results show that the improved algorithm has an accuracy rate of 90.7% on the KITTI data set. Compared with YOLOv4, the parameters of the proposed MobileYOLO model are reduced by 52.11 M, the model size is reduced to one-fifth, and the detection speed is increased by 70%.

## 1. Introduction

In recent years, deep learning has been widely used in various fields, and computer vision has gradually become a leader among them. Real-time object detection in driving scenes has become a hot research topic in the field of computer vision. As one of the most basic parts of autonomous driving, object detection collects real-time environmental information for vehicles to ensure safety and provide correct planning decisions [1]. Object detection algorithms based on deep learning have shown unique advantages in the field of autonomous driving. They can obtain high detection accuracy under the premise of fewer computing resources, thus becoming an indispensable method in autonomous driving systems [2].

At present, the commonly used object detection frameworks are divided into two categories. One is the Region-Convolutional Neural Network (R-CNN) series [3,4,5,6] of two-stage object detection algorithms proposed by Ross Girshick et al. The two-stage object detection algorithm has high detection accuracy, but the detection speed is slow, and the model is large, which makes it difficult to meet real-time detection in autonomous driving scenarios. The other category is one-stage object detection algorithms such as Single-Shot MultiBox Detector (SSD) [7] and You Only Look Once(YOLO) series [8,9,10,11] proposed by Joseph Redmon et al. The detection speed of the one-stage algorithm can meet the real-time requirements in driving scenarios, but its detection accuracy is lower than that of the two-stage detector. In recent years, many one-stage algorithms with high model efficiency have been applied to driving scenarios, such as YOLOv3, Centernet [12], Retinanet [13] and so on. The prerequisite for object detection algorithms in autonomous driving applications is a high processing speed. Although the above methods can perform real-time detection, it is still very difficult to apply in autonomous driving at the cost of low-resolution input. Therefore, the trade-off between detection speed and accuracy of the algorithm is still one of the most important issues in practical applications.

Furthermore, most of the previous studies only focus on one or some specific resource requirements, while a large number of practical applications usually have different resource constraints. Autonomous driving computing platforms have limited computing resources and need to handle multiple sensors and computing tasks, such as detection, tracking, and decision-making. Therefore, the latest trend in neural network design should be to explore portable and efficient network architectures, and detection algorithms applied to in-vehicle platforms need to have a relatively small proportion of memory and computing resources. Therefore, it is necessary to design a lightweight and efficient object detection model.

In the field of autonomous driving, the main detected objects are divided into two categories: stationary objects and moving objects. Stationary objects include traffic signs, traffic lights, obstacles, etc., and moving objects include vehicles, pedestrians, non-motorized vehicles, etc. Among them, the detection of moving objects is particularly important. It has the following difficulties: (1) the object to be detected has different degrees of occlusion; (2) the detection effect for small objects is not good; (3) the detection speed and detection accuracy are often not satisfied at the same time. Therefore, whether the above problems can be solved directly affects the safety performance of autonomous driving.

Because of the above problems, this paper combines the advantages of one-stage and two-stage object detection, trade-offs the detection accuracy and detection speed, and proposes a lightweight and efficient object detection model called mobileYOLO, which can meet the requirements of detection accuracy and speed in autonomous driving scenarios at the same time. The main contributions are as follows:

(1) Introducing the MobileNetv2 network to reconstruct the feature extraction network, which greatly reduces the number of model parameters;

(2) Replacing part of standard convolution with depthwise separable convolution in PAnet and the head network, which further reduces the number of model parameters;

(3) The ECA module is introduced to enhance the representation ability during feature fusion, and the SSH context module is introduced to improve the receptive field and improve the small object detection ability.

## 2. Related Work

### 2.1. Object Detection Algorithm Based on Deep Learning

Object detection based on deep learning is mainly divided into one-stage and two-stage object detection algorithms. The two-stage object detection algorithm preliminarily selects the location of the object through a region proposal network (RPN) [5] and then classifies and predicts the selected region. The representative two-stage object detection algorithms include Faster R-CNN [5] and Mask R-CNN [6]. Although this type of object detection algorithm has high precision, it has a large amount of computation, so it is difficult to meet real-time requirements.

The one-stage object detection algorithm can perform classification prediction and regression prediction on the object at the same time, which has the advantages of less computational complexity and a fast inference speed. SSD and YOLO series are typical one-stage object detection algorithms. SSD [7] detects objects by forming a multi-scale feature map with convolution layers of different sizes. DSSD [14] adds a deconvolution layer to the SSD model, which improves the detection effect of small objects while maintaining the detection speed.

The YOLO [8] series of algorithms divide the input object into a specific number of grids and then determine the position of the bounding box of each grid cell object and the category of the object. The YOLOv2 [9] runs k-means clustering on the training set BBoxes to automatically find nine precise anchors. The YOLOv3 [10] employs Darknet53 as a new Convolutional Neural Network (CNN) backbone to extract features from input images and introduces a feature pyramid structure to achieve multi-scale Feature fusion. YOLOv4 [11] follows the head structure in YOLOv3, uses CSPDarknet53 [15] as the new backbone network, and adds Spatial Pyramid Pooling (SPP) [16] and Path Aggregation Network (PAN) [17] structures to the Neck part to improve the overall performance of the model.

YOLOv4 is an excellent one-stage object detection algorithm. In the NVIDIA GTX 1080Ti environment, its running speed can reach 62FPS, but it is still difficult to run in real-time on resource-limited autonomous driving devices.

### 2.2. Model Lightweight Methods

In order to ensure accuracy in computer vision tasks, networks with very deep layers are often used, which leads to a large number of network parameters and a large amount of calculation in the forward derivation process, such as ResNet [17], Inception series [18,19,20], etc. A large number of parameters make these networks take up a big amount of storage space, both in predictive deployment and training. Therefore, how to reduce the number of parameters and computation as much as possible on the premise of ensuring the high performance of the network has become a research hotspot, which is called model lightweighting. In computer vision, the compression model is most often used to reduce the weight of the model, such as:

(1) Efficient network structure: Design a lightweight network to reduce redundant parameters in the model. For example, SqueezeNet [21] application module convolution; MobileNet [22,23,24] pursues the ultimate network compression effect and designed a deep separable convolution; ShuffleNet [25,26] inherits the residual structure while using grouped convolution and deep separable convolution, which greatly accelerates the forward derivation process of ResNet. These sophisticated and high-efficiency network structures catch up with mainstream networks in accuracy, and the number of parameters and calculations are much smaller.

(2) Network pruning: There are often a large number of redundant connections in the network, and most of them do not play a role in the training and prediction processes of the network. The operation of network pruning is to find these unnecessary connections and remove them, making the neural network itself sparse, thereby reducing the number of network calculations and model storage.

(3) Knowledge distillation: This operation trains a large teacher network in advance and then uses another training method to train a small student network. In order to obtain a small model that is easier to deploy, this small model inherits the knowledge of the previous large model.

### 2.3. Object Detection in Autonomous Driving Scenarios

Object detection algorithms based on deep learning are mainly used in vehicle detection, pedestrian detection, and traffic sign detection in autonomous driving scenarios. Wang et al. proposed a method that can effectively detect small vehicles in traffic scenes using multiscale feature fusion and focal loss [27]; Chen et al. used group convolution to design a lightweight detection network for autonomous driving scenarios [28]; Liu et al. proposed an efficient pedestrian detection algorithm based on SSD to solve the problem of missed detection in dense crowds [29]; finally, Z Liu et al. proposed a deconvolution region-based convolutional neural network to solve the problem of small traffic sign detection [30].

Different from previous work, this paper proposes an efficient object detection model for autonomous driving scenarios, which guarantees very accurate detection while meeting real-time conditions and trade-off the accuracy and speed. It has a good performance in the detection of pedestrians and vehicles in autonomous driving scenarios.

## 3. Related Models and Network Design

### 3.1. Yolo Object Detection Model

**Figure 1:**
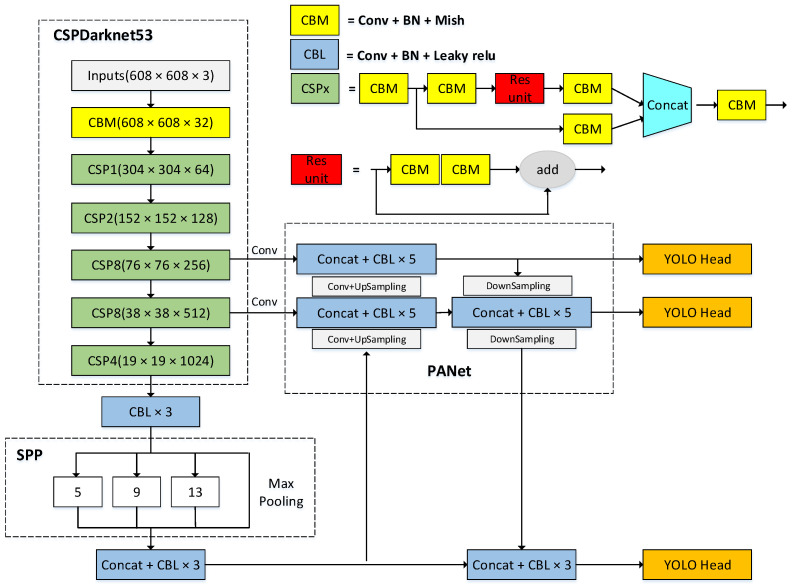
YOLOv4 model structure.

As a classic one-stage object detection network, YOLOv4 can directly generate predicted object classification and bounding boxes, which greatly improves the speed of object detection. Its structure is shown in Figure 1.

The YOLOv4 network model consists of three major parts: backbone network, neck network, and head network. The backbone network is the CSPDarknet53 feature extraction network, which is composed of five modules CSP1–CSP5. Each module is formed by alternately stacking CSPx modules and CBM or CBL modules. The x in CSPx represents the number of CSP blocks. The CSP first divides the input into two branches. One branch first performs the CBM operation, passes through the residual structure, and then performs the CBM operation again; the other branch directly performs the CBM operation. The two branches are output after the concat operation. CBM represents convolution, Batch Normalization (BN), and Mish activation function; CBL represents convolution, BN, and Leaky ReLU activation function. YOLOv4 uses the CSPnet structure in the backbone network. The other side of the Res unit stack is processed by CBM to form a large residual side, which enhances the learning ability. In the CSPnet structure, the Mish activation function is introduced to replace the Leaky ReLU activation function. The Mish function has the characteristics of no upper bound, lower bound, non-monotonic, infinite-order continuity, and smoothness, which help the model realize regularization and stabilize the network gradient.

The *Mish* function expression is:

(1) $$Mish=x\tanh(\ln\left(1+e^{x}\right))$$

where *x* is the input value, tanh is the hyperbolic tangent function, and ln is the logarithmic function with the constant *e* as the base.

After the CSPDarknet53 backbone network, we obtain 76 × 76 × 256 (feature layer P3), 38 × 38 × 512 (feature layer P4), and 19 × 19 × 1024 feature layers. Then, the 19 × 19 × 1024 feature layer is subjected to maximum pooling of different sizes of 13 × 13, 9 × 9, and 5 × 5, 1 × 1. Finally, the feature layer P5 is obtained through the concatenate operation and then through the concatenate operation to obtain the feature layer P5.

The SPP structure greatly increases the receptive field and obtains more contextual features. The three feature layers of P3, P4, and P5 pass through the PANet, and after the upsampling fusion from the bottom to the top, the three feature layers are then enhanced by downsampling from top to bottom. It greatly shortens the information propagation path and utilizes the precise positioning information of low-level features.

The loss function Loss of YOLOv4 includes regression loss function Loss (coord), confidence loss function Loss (conf), and classification loss function Loss (cls). The loss function formula is as follows:

(2) Loss=Loss(coord)+Loss(conf)+Loss(cls)

(3) $$Loss\left(coord\right)=\lambda_{coord}\sum_{i=0}^{K\times K}\sum_{j=0}^{M}I_{ij}^{obj}(2-w_{i}\times h_{i})$$

(4) $$\begin{array}{c} Loss\left(conf\right)=-\sum_{i=0}^{K\times K}\sum_{j=0}^{M}I_{ij}^{obj}[\overset{\wedge }{C_{i}}\mathrm{lg}C_{i}+(1-\overset{\wedge }{C_{i}})\mathrm{lg}\left(1-C_{i}\right)]\\ -\lambda_{noobj}\sum_{i=0}^{K\times K}\times \sum_{j=0}^{M}I_{ij}^{noobj}\left[\overset{\wedge }{C_{i}}\mathrm{lg}C_{i}+\left(1-\overset{\wedge }{C_{i}}\right)\mathrm{lg}\left(1-C_{i}\right)\right] \end{array}$$

(5) $$\begin{array}{c} Loss\left(cls\right)=-\sum_{i=0}^{K\times K}I_{ij}^{obj}\sum_{c\in classes}[\overset{\wedge }{p_{i}}\left(c\right)\mathrm{lg}p_{i}\left(c\right)\\ +\left(1-\overset{\wedge }{p_{i}}\left(c\right)\right)\mathrm{lg}\left(1-p_{i}\left(c\right)\right)] \end{array}$$

where *K* is the grid size, *I* represents the i-th square of the feature map, *j* represents the j-th prediction box predicted by the square, *w* and *h* represent the width and height of the ground truth, respectively, and the subscripts obj and noobj, respectively, indicate the presence and absence of objects in the i-th square. $C_{i}$ and $\overset{\wedge }{C_{i}}$ represent the categories of prediction and real boxes, respectively, $P_{i}\left(c\right)$ is the confidence of the predicted object, $\overset{\wedge }{P_{i}}\left(c\right)\;$ is the confidence of the actual object, and $\lambda_{coord}$ and $\lambda_{noobj}$ are penalty coefficients.

### 3.2. Lightweight YOLOv4 Network Design

**Figure 2:**
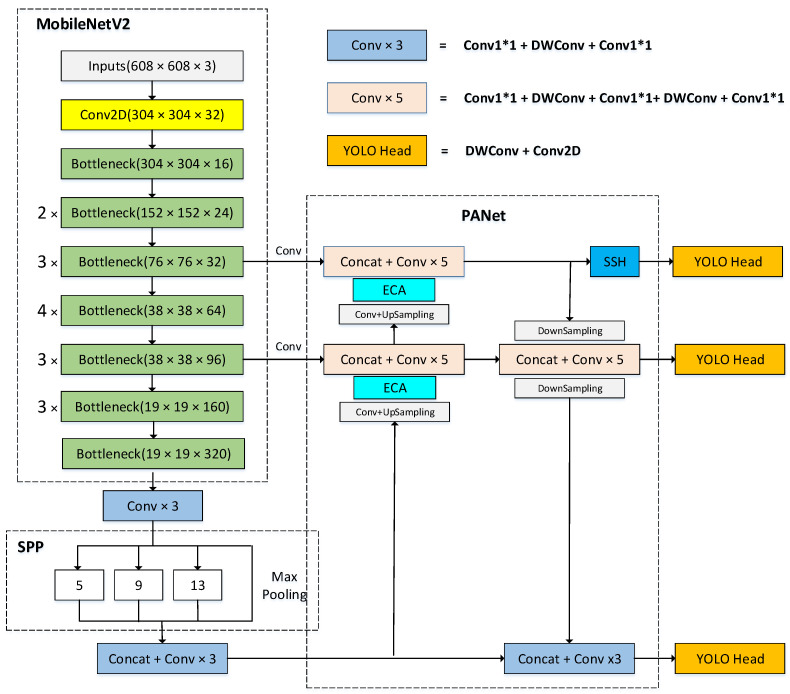
MobileYOLO model structure.

Based on YOLOv4, this paper proposes the MobileYOLO algorithm. The overall structure is shown in Figure 2, with some lightweight improvements on MobileYOLO.

First, replacing the CSPdarknet53 backbone network in YOLOv4 with the Mobilenetv2 lightweight backbone network reduces the number of model parameters and use Depthwise Separable Convolution instead of traditional convolution in the feature fusion network and the detection head network further reduces the number of parameters. Then, a lightweight attention module ECANet is added to the feature fusion network to enhance effective features, suppress useless features, and improve the characterization ability during feature fusion. Finally, the SSH context module is added to the 76 × 76 small object feature map branch to increase the receptive field and improve the detection ability of small objects. The other parts are consistent with YOLOv4.

#### 3.2.1. Depth Separable Convolution

**Figure 3:**
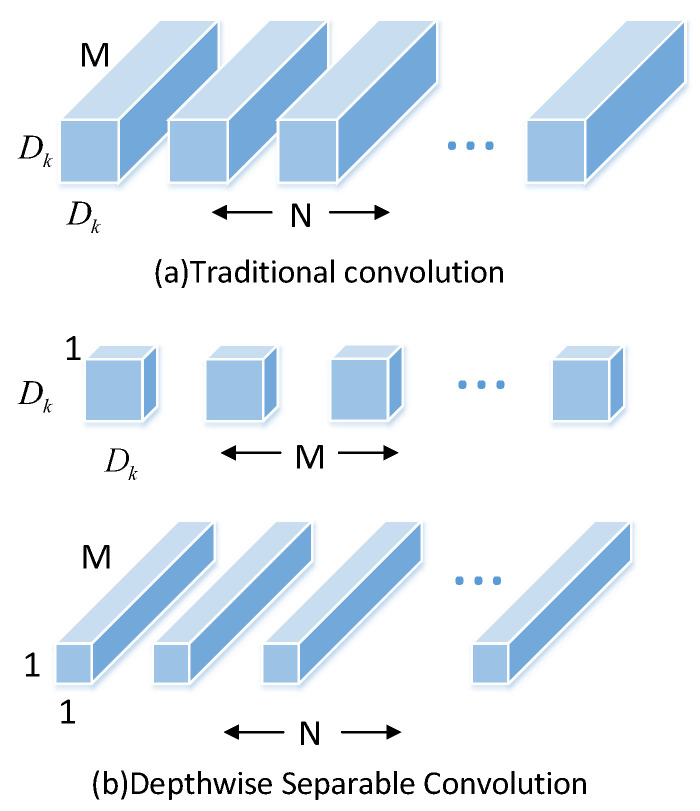
The difference between traditional convolution and depthwise separable convolution.

The depth separable convolution consists of the depthwise convolution (DWConv) operation and the pointwise convolution (PWConv) operation. The difference from the traditional convolution is shown in Figure 3. The depthwise separable convolution performs a convolution operation with a single convolution kernel size of 3 × 3 on each channel dimension of the input and then combines the features of all input channel dimensions by performing a standard convolution operation with a convolution kernel size of 1 × 1 improved features. The calculation amount of the standard convolution is *k* × *k* times different from the depth separable convolution (*k* is the size of the convolution kernel), which greatly reduces the number of model calculations without sacrificing accuracy.

MobileYOLO not only uses the backbone network MobileNetV2 with deep separable convolution but also replaces part of the traditional convolution in the neck network and head network of the model with deep separable convolution (see Figure 2).

#### 3.2.2. Mobilenetv2 Network Structure

This paper uses the MobileNetV2 as the improved backbone network to complete the task of extracting object features in autonomous driving scenarios. MobileNetV2 continues to use the deep separable convolution in the MobileNetV1 network, so it can greatly reduce model parameters. Compared with MobileNetV1, MobileNetV2 introduces an inverted residual module containing linear bottleneck blocks, which effectively improves the accuracy of image classification and detection tasks applied on the mobile terminal.

Performing the ReLU activation function operation on the low-dimensional convolutional layer can easily cause the loss of characteristic information, while performing the ReLU activation function operation on the high-dimensional convolutional layer can reduce the loss of information. Therefore, the MobileNetV2 network does not use an activation function in the low-dimensional convolutional layer of the bottleneck block and instead replaces it with a linear bottleneck layer. In the high-dimensional convolution layer, the ReLU6 activation function proposed by MobileNetV1 is used to prevent the nonlinear layer from destroying too much feature information.

**Figure 4:**
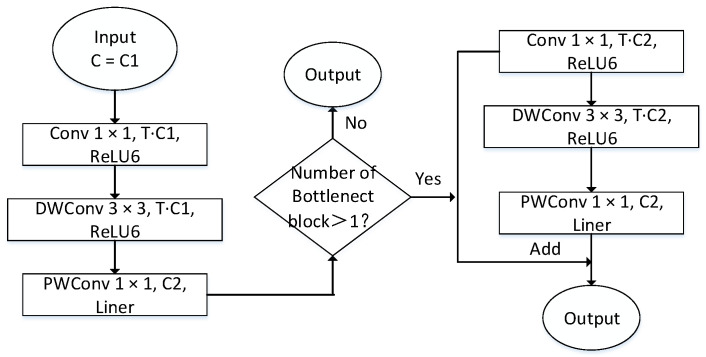
Bottleneck block structure of MobileNetV2.

The bottleneck block structure of the MobileNetV2 network is shown in Figure 4. The inverted residual module is mainly used to promote the effective transmission of multi-layer feature information and enhance the feature extraction capability of the network. The inverted residual module first performs channel expansion through convolution with a convolution kernel size of 1 × 1 and then uses a deep convolution with a convolution kernel size of 3 × 3 to extract features, and finally, the channel dimension is compressed by the point-by-point convolution with the convolution kernel size of 1 × 1 in the linear bottleneck layer. When the bottleneck block used in MobileNetV2 is greater than 1, the bottleneck block contains the inverted residual module.

Note: C is the channel dimension, C1 is the input channel dimension of the bottleneck block, C2 is the output channel dimension of the bottleneck block, T is the channel dimension expansion factor, Conv 1 × 1 is a standard convolution operator with convolution kernels of 1 × 1, ReLU6 is the activation function, and Liner represents the activation function is not used in the convolution operator.

### 3.3. Eca Lightweight Attention Module

In the process of feature fusion, part of the effective feature information will inevitably be lost. If the attention mechanism is introduced to improve the feature fusion module, the algorithm can obtain higher-quality detection feature maps, which can enhance the effective feature weights while suppressing invalid features to improve the detection information loss caused by the feature fusion process.

Comparing the performance of different attention modules on the same network (ResNet), as shown in Table 1, the TOP-1 accuracy rate indicates the accuracy rate of whether the first-ranked category matches the actual result when different algorithms are tested on the ImageNet data set.

It can be seen from Table 1 that compared to the original network without the attention mechanism, the SE module, the CBAM module, and the AA module increase the parameters by 4.52 M, 4.52 M, and 2.91 M, respectively. After adding the ECA module, the parameters and the amount of calculation hardly increase. Combined with the TOP-1 accuracy test results, it can be seen that ECA can achieve higher detection accuracy while introducing minimal computational burden, with lightweight characteristics and efficient operation.

The SE [31] module uses global average pooling and weighting factors to enhance the feature expression in the channel dimension, and high-quality detection is particularly conducive to improving the accuracy of object detection. CBAM uses global maximum pooling and global average pooling to obtain feedback of each pixel and salient feature point in the feature map and completes feature enhancement in space and channel dimensions. ECA uses one-dimensional convolution to enhance effective feature weights, and only introduces very few parameters and calculations. Compared with other attention modules, the detection effect is significantly improved, and it has better lightweight application characteristics.

**Figure 5:**
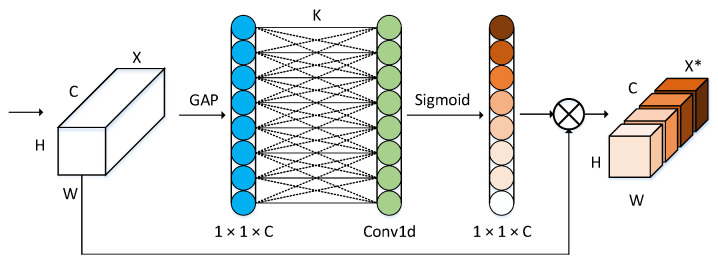
The structure of ECA.

Autonomous driving scenarios include pedestrians and vehicle objects of different scales. Traditional attention mechanisms often use features to reduce dimensionality, which often loses key information for small-scale object detection. However, fully connected layers require a large number of parameters and calculations, unable to make effective use of the correlation between channels. ECA [32] first performs feature integration, introduces global average pooling, and obtains important detection information of each feature map. The output feature dimension is *C* × 1 × 1, and *C* is the number of feature channels. ECA directly uses the one-dimensional convolution with the convolution kernel number *k* to obtain feature information to avoid information loss caused by the dimensionality reduction process. The convolution kernel scale *k* is adaptively determined by the channel parameters and does not need to be cross-validated for manual tuning, such as Formula (6). Then, it used the Sigmoid activation function to output a new weight parameter with dimension *c*. The weight parameter better reflects the importance and correlation of different channels. Finally, the new weight parameter is multiplied with the input feature map, and the weights of different channel features are redistributed to better suppress invalid features and enhance the weight of effective features. Compared with attention modules, such as SENet, the ECA module is lighter and more efficient.

(6) $$k=\varphi \left(c\right)={\left|\frac{\log_{2}\left(c\right)}{\gamma }+\frac{b}{\gamma }\right|}_{odd}$$

where *c* is the number of characteristic channels, ${\left|*\right|}_{odd}$ is the nearest odd number; γ = 2, *b* = 1. The structure of ECA is shown in Figure 5.

### 3.4. Ssh Context Module

In a two-stage detector, contextual information is usually integrated by expanding the detection window of the proposals. Since anchors are classified and regressed in a convolutional manner, a larger convolution kernel is used to expand the receptive field, which is similar to increasing the window size around proposals in a two-stage detector.

**Figure 6:**
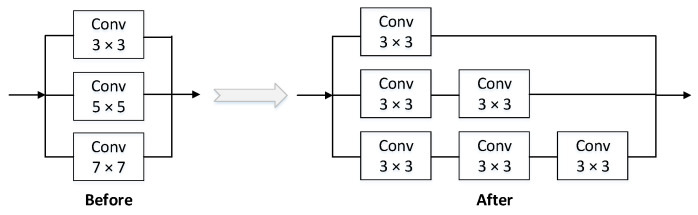
The structure of SSH.

Unlike the two-stage detector, SSH [33] mimics the operation of contextual information enhancement by superimposing simple convolution. In the context module, multiple 3 × 3 size convolutions are used to simulate 5 × 5 and 7 × 7 convolution kernels. This method increases the receptive field corresponding to the asynchronous length of the corresponding detection layer and also replaces the larger convolution in this way. The kernel then reduces the number of parameters, as shown in Figure 6. (Note: The large convolution kernel can obtain a larger receptive field, but the number of parameters is larger. Using some smaller convolution kernel layers to stack up can achieve the same effect as the large receptive field while reducing the number of model parameters.)

If the SSH context module is added to all three outputs of PAnet, the number of parameters will be increased, resulting in a waste of computing resources. Therefore, this experiment only adds the SSH module to the output of 76 × 76 size (shallow features contain more position information and are suitable for detecting small objects) to improve the characterization ability of small objects.

## 4. Experiments and Results

### 4.1. Data Set and Experimental Environment

The KITTI [34] data set is currently the world’s largest computer vision algorithm evaluation data set in autonomous driving scenarios. It contains real image data collected in urban, rural, and highway scenarios. Each image can contain up to 15 cars and 30 pedestrians. There are various degrees of occlusion and truncation, which is convenient to verify the effectiveness of MobileYOLO in this paper. The KITTI data set contains 7481 training sets and 7518 test sets. There are eight types of objects, namely Car, Van, Truck, Pedestrain, Person (sitting), Cyclist, Tram, and Misc. In order to facilitate the experiment, this experiment merges the three categories of Pedestrain, Person (sitting), and Cyclist into the same category, named Person. Furthermore, the 7481 training set is divided into the training set and the test set of this experiment according to the ratio of 7:3.

This paper uses the Pytorch deep learning framework to conduct experiments under the Ubuntu 16.04 system. The machine configuration used in the experimentis as follows: the processor is Intel(R) Core(TM) i7-7800X; the memory is 32 G; and the graphics card is 2 NVIDIA 1080Ti (11 GB). The software environment used is Pytorch1.7.0, Python3.6, CUDA10.1, cuDNN7.3.1.

The model training in this paper adopts the transfer learning method. First, the pre-training weights are loaded onto the VOC07+12 data set to accelerate the convergence of the model training, and a two-stage training method is adopted.

The first stage of training freezes the backbone network to prevent excessive randomness in the initial training period and network fluctuations destroying the initial weight. During training, set the batchsize to 64, the learning rate to 0.001, the momentum to 0.9, and the attenuation coefficient of the weight to 0.0005. The input resolution is 608 × 608, and a total of 20 epochs are trained.

In the second stage of unfreezing the backbone network during training, set the batchsize to 32, the learning rate to 0.0001, the momentum to 0.9, and the attenuation coefficient of the weight to 0.0005. The input resolution is 608 × 608, and a total of 60 epochs are trained.

**Figure 7:**
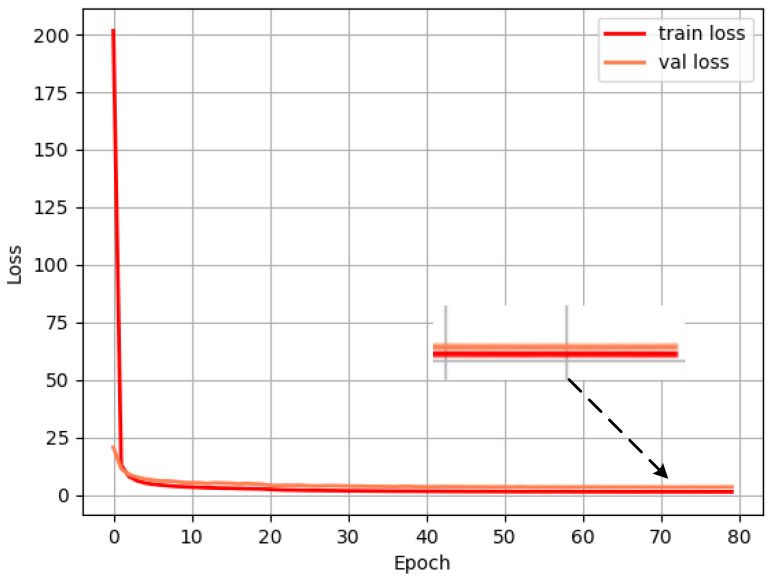
Loss convergence of MobileYOLO.

The loss value is recorded for every iteration of an epoch, and the loss convergence of MobileYOLO on the KITTI data set is shown in Figure 7.

The results show that the training loss curve and the validation loss curve continue to decline, and the model does not diverge or overfit during the entire training process, which illustrates the effectiveness of the MobileYOLO model structure. After iterating to 65 Epochs, the loss stabilizes around 1.55, and the model reaches the optimal state.

### 4.2. Evaluation Index

In order to evaluate the effectiveness of the proposed method, the number of frames per second (FPS) is used as the speed evaluation index of the model, and the mean average accuracy (mAP) is used as the evaluation index of model accuracy, which represents the average value of each type of *AP* in the data set.

*AP* is measured from the two perspectives of precision and recall and is an intuitive evaluation standard for evaluating the accuracy of the detection model. *F*1 is the harmonic average of precision and recall and is an index used to measure the accuracy of the model.

The calculation formulas for precision rate, recall rate, *F*1 value, and *AP* are as follows

(7) $$\mathrm{Precision}=\frac{TP}{TP+FP}$$

(8) $$\mathrm{Recall}=\frac{TP}{TP+FN}$$

(9) F1=2×Precision×RecallPrecision+Recall

(10) $$AP=\int_{0}^{1}P(R)dR$$

Among them, *TP* is the number of true positive samples; *FP* is the number of false positive samples; *FN* is the number of false negative samples. Using recall rate and accuracy rate as the abscissa and ordinate, respectively, draw the P–R curve. The area enclosed by the curve is the value of *AP*. The larger the area, the higher the detection accuracy.

### 4.3. Analysis of Experimental Results

#### 4.3.1. Comparison of YOLOv4 and MobileYOLO

After training the MobileYOLO network, we use the model to test and obtain the experimental results. Table 2 is a comparison of the experimental results of MobileYOLO and YOLOv4.

From the experimental results in Table 2, it can be seen that in the four categories of Truck, Car, Van, and Tram, the accuracy of MobileYOLO is close to YOLOv4. In the category of Person, it has dropped by 6% compared to YOLOv4. It is because the data are concentrated with dense crowds and severe occlusion, which leads to an increase in the missed detection rate. Overall, MobileYOLO’s precision, recall, F1-score, and mAP are close to YOLOv4, dropping by 1%, 6%, 3%, and 2.6%, respectively, but FPS improved by nearly 70%. At the expense of a small amount of accuracy, the detection speed is greatly improved.

**Figure 8:**
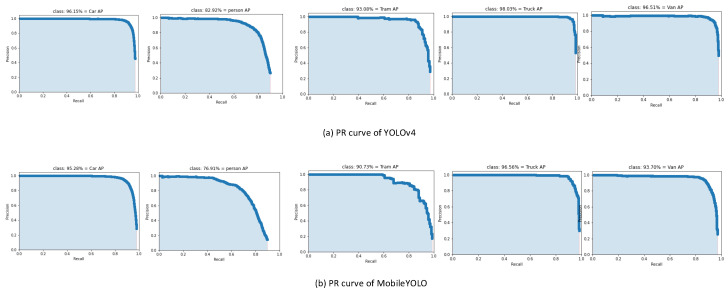
The P–R curves of the detection results.

Figure 8 are the P–R curves of the detection results of YOLOv4 and MobileYOLO in the KITTI data set, respectively. The detection performance of the algorithms can be intuitively compared through the P–R curve. The results show that MobileYOLO is very close to YOLOv4 in detection accuracy.

**Figure 9:**
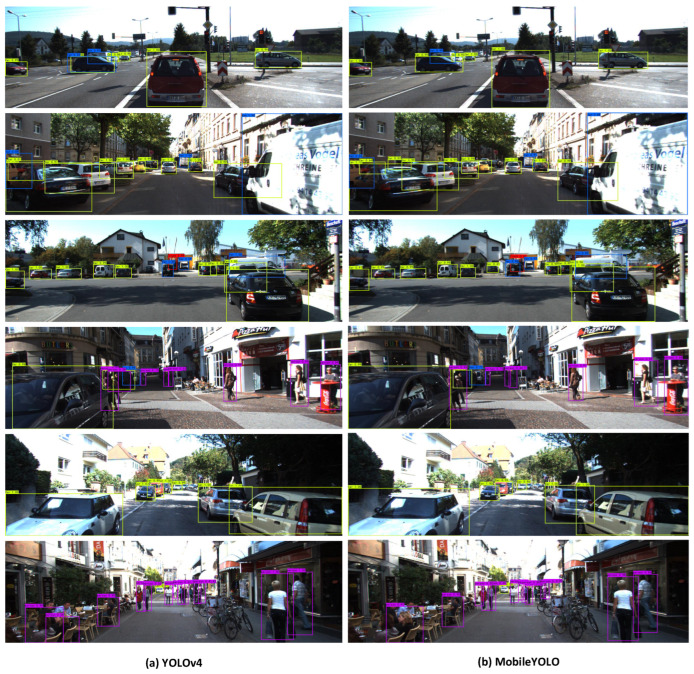
The visualization of results.

Figure 9 is a visual comparison of the pedestrian and vehicle detection results of YOLOv4 and MobileYOLO in autonomous driving scenarios, respectively. Each detection box in the figure represents the final prediction result, and the top of the detection box represents the category and confidence of its detection. The boxes of yellow, blue, red, and purple represent the detection of Car, Van, Truck, and Person, respectively. From the visualization results, the proposed MobileYOLO has a good detection effect, which is comparable to YOLOv4, without too many false detections and missed detections, and has good applicability in autonomous driving scenarios.

#### 4.3.2. Ablation Experiment

The ablation experiment is a commonly used experimental method in the field of deep learning, which is mainly used to analyze the influence of different network branches on the entire model. In order to further analyze the influence of the MobileNetV2, depth separable convolution, the ECA attention module, and the SSH context module introduced in this paper on the YOLOv4 model, ablation experiments were carried out. The specific results are shown in Table 3, where “✓” represents the corresponding improvement method.

In experiments (1) and (2), the introduction of MobileNetV2 and deep separable volume actively reduces the number of model parameters, which improves the detection speed but also reduces the model detection accuracy. In experiments (3)–(6), the introduction of the ECA module and the SSH module were compared experimentally, and the results showed that adding a small number of parameters can increase the mAP by 2.1%. It can be seen that the improvements made in this paper for YOLOv4 are meaningful, achieving a trade-off between detection speed and accuracy and having better applicability in autonomous driving scenarios.

The result shows that although the MobileYOLO reduces the mAP by 2.6% compared to YOLOv4, it also greatly reduces the number of model parameters of 52.11 M, the model size is reduced to more than one-fifth of the original, and FPS increased by 70%.

#### 4.3.3. Comparison of Different Algorithms

In order to further verify the effectiveness and scientificity of the algorithm in this paper, the proposed MobileYOLO is compared with the current mainstream one-stage object detection algorithm. The comparison results are shown in Table 4.

It can be seen from Table 4 that the mAP and FPS of MobileYOLO on the KITTI data set are greatly improved compared to SSD300, YOLOv3, and RetinaNet, and the model parameters and weights are lower than the above algorithms. MobileYOLO’s FPS is 15.5 lower than YOLOv4-tiny, but it is 8.1% higher than YOLOv4 on mAP. Similarly, it is 2.6% lower than YOLOv4 on mAP but has an increase of 32.7in FPS, and the amount of model parameters and the size of the weight is greatly reduced. In general, the MobileYOLO is more cost-effective, combines the advantages of YOLOv4 and YOLOv4-tiny, trades off detection speed and accuracy to a certain extent, and is more suitable for autonomous driving scenarios.

### 4.4. Natural Scene Video Detection Experiments

**Figure 10:**
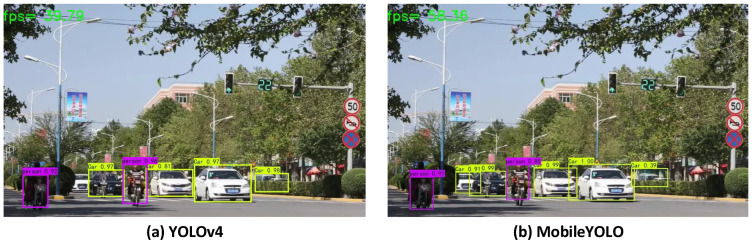
Video detection effect.

In order to verify the effectiveness of the object detection algorithms in natural scenes, a test experiment was carried out on the video. The experimental results are shown in Figure 10. The video is downloaded from the Internet, and the video size is 800 × 480. The purple and yellow detection boxes represent the detection of people and cars, respectively. It can be seen that the proposed MobileYOLO can effectively detect the objects in the video as a whole. Compared with YOLOv4, the FPS is nearly 20 higher, which further proves the superiority of the algorithm in the autonomous driving scenario.

## 5. Conclusions

Based on the YOLOv4 algorithm, this paper proposes a real-time object detection algorithm. The experimental results show that the the proposed MobileYOLO has an accuracy rate of 90.7% on the KITTI data set. Compared with YOLOv4, the parameters of the proposed MobileYOLO model are reduced by 52.11 M, the model size is reduced to one-fifth, and the detection speed is increased by 70%, which reduces computing power requirements and training costs. It is beneficial to the deployment of embedded devices. However, the MobileYOLO proposed in this paper is a method of supervised learning, which requires a large number of samples for training. Reducing the experimental sample size and using semi-supervised methods should be focus of future research.

## Figures and Tables

**Table 1:** Performance indicators of different attention mechanisms.

Method	Params/M	FlOPs/G	TOP-1 Accuracy/%
ResNet-50	42.49	7.34	76.83
+SE	47.01	7.35	77.62
+CBAM	47.01	7.35	78.49
+AA	45.40	8.05	78.70
+ECA	42.49	7.35	78.65

**Table 2:** The experimental results of YOLOv4 and MobileYOLO.

Method	Truck	Car	Van	Tram	Person	Precision	Recall	F1	mAP@50	FPS
YOLOv4	0.980	0.962	0.965	0.931	0.829	0.92	0.88	0.89	0.933	47.6
MobileYOLO	0.966	0.953	0.937	0.907	0.769	0.91	0.82	0.86	0.907	80.3

**Table 3:** Results of ablation experiment.

Method	MobileNetV2	DWConv	ECA	SSH	mAP	Params(M)	Volume(MB)	FPS
YOLOv4(0)					0.933	64.36	245.5	47.6
YOLOv4(1)	✓				0.915	39.1	98.4	68.4
YOLOv4(2)	✓	✓			0.886	12.11	46.2	81.7
YOLOv4(3)			✓		0.942	64.36	245.7	47.2
YOLOv4(4)				✓	0.940	64.45	246.1	47.1
YOLOv4(5)	✓	✓	✓		0.897	12.11	46.3	81.0
MobileYOLO(6)	✓	✓	✓	✓	0.907	12.25	46.7	80.3

**Table 4:** Comparison of other algorithm results.

Method	Backbone	Input Size	mAP	Params (M)	Volume (MB)	FPS
SSD300 [7]	VGG-16	300 × 300	0.835	24.26	92.2	48.2
YOLOv3 [10]	Darknet-53	608 × 608	0.874	61.53	234.7	42.1
RetinaNet [13]	ResNet-50	500 × 500	0.887	37.23	165.4	36.5
YOLOv4-tiny	CSPdarknet-53	608 × 608	0.826	6.28	22.4	95.8
YOLOv4 [11]	CSPdarknet-53	608 × 608	0.933	64.36	245.5	47.6
MobileYOLO(Ours)	MobileNetV2	608 × 608	0.907	12.25	46.7	80.3

## Data Availability

The data presented in this study are available on request from the corresponding author.
